# Candidate Regulators of Dyslipidemia in Chromosome 1 Substitution Lines Using Liver Co-Expression Profiling Analysis

**DOI:** 10.3389/fgene.2019.01258

**Published:** 2020-01-09

**Authors:** Fuyi Xu, Maochun Wang, Shixian Hu, Yuxun Zhou, John Collyer, Kai Li, Hongyan Xu, Junhua Xiao

**Affiliations:** ^1^College of Chemistry, Chemical Engineering, and Biotechnology, Donghua University, Shanghai, China; ^2^Department of Genetics, Genomics, and Informatics, University of Tennessee Health Science Center, Memphis, TN, United States; ^3^Department of Gastroenterology and Hepatology, University of Groningen and University Medical Center Groningen, Groningen, Netherlands; ^4^Department of Pediatrics, University of Tennessee Health Science Center, Memphis, TN, United States; ^5^Department of Biostatistics and Epidemiology, Medical College of Georgia, Augusta University, Augusta, GA, United States

**Keywords:** plasma lipid, Chr 1 substitution lines, gene network, genome wide association studies, candidate gene

## Abstract

Dyslipidemia is a major risk factor for cardiovascular disease. Although many genetic factors have been unveiled, a large fraction of the phenotypic variance still needs further investigation. Chromosome 1 (Chr 1) harbors multiple gene loci that regulate blood lipid levels, and identifying functional genes in these loci has proved challenging. We constructed a mouse population, Chr 1 substitution lines (C1SLs), where only Chr 1 differs from the recipient strain C57BL/6J (B6), while the remaining chromosomes are unchanged. Therefore, any phenotypic variance between C1SLs and B6 can be attributed to the differences in Chr 1. In this study, we assayed plasma lipid and glucose levels in 13 C1SLs and their recipient strain B6. Through weighted gene co-expression network analysis of liver transcriptome and “guilty-by-association” study, eight associated modules of plasma lipid and glucose were identified. Further joint analysis of human genome wide association studies revealed 48 candidate genes. In addition, 38 genes located on Chr 1 were also uncovered, and 13 of which have been functionally validated in mouse models. These results suggest that C1SLs are ideal mouse models to identify functional genes on Chr 1 associated with complex traits, like dyslipidemia, by using gene co-expression network analysis.

## Introduction

Plasma lipid levels of total cholesterol (CHOL), high-density lipoprotein cholesterol (HDL-C), Low-density lipoprotein cholesterol (LDL-C), and triglycerides (TG), are major contributors to cardiovascular diseases (Kathiresan et al., 2007). Current evidence demonstrates that both environmental and genetic factors contribute to these lipid levels. Therefore, discovery of the genetic regulators would be beneficial to determine individual susceptibility to dyslipidemia and eventually for developing gene therapies.

Recent genome wide association studies (GWAS) in humans have linked hundreds of genetic loci to plasma lipid metabolism, including genes *APOE*, *PCSK9*, *CETP*, *LIPC*, *LPL*, and *APOA5* (Willer et al., 2013a; Helgadottir et al., 2016). Furthermore, several rare variants have been uncovered with next generation sequencing technology (Natarajan et al., 2018). Although significant achievements have been made, the identified genetic loci only explain a small portion of the phenotypic variance, suggesting most of the genetic regulators remain unknown.

Mouse models have been widely used for deciphering regulatory genes of quantitative traits. Hundreds of genetic loci have been identified through quantitative trait loci (QTL) mapping in F2 or backcross mouse populations (http://www.informatics.jax.org/). However, it’s challenging to identify causative genes within QTLs. During the past decade, mouse genetic reference populations, such as BXD recombinant inbred strains (Wang et al., 2016), Collaborative Cross (Consortium, 2012), Hybrid Mouse Diversity Panel (Ghazalpour et al., 2012), and chromosome substitution strains (CSSs) (Nadeau et al., 2000), have significantly accelerated the precise QTL mapping and functional gene identification through improved mapping power and resolution (Buchner and Nadeau, 2015). CSSs, which typically involve two inbred strains with significant phenotypic differences, are a panel of inbred strains by backcrossing the donor and recipient parents over 10 generations. The final panel contains the entire genome information of both strains, and each CSS carries one intact donor chromosome in the genetic background of the recipient strain. Therefore, any phenotypic differences between CSSs and recipient strain can be attributed to the substituted chromosome. This allows for easy detection of genes for multi-genic traits and quick identification of QTLs through linkage analysis in F2 population and fine mapping with congenic strains. Previously, we proposed a novel strategy of developing a Chr 1-specific CSS substitution line (C1SL) to dissect the complex traits. With this strategy, Chr 1 of the recipient strain C57BL/6J (B6) was replaced by different wild mice individually (Xiao et al., 2010; Xu et al., 2016). Compared to CSSs, C1SLs are suitable for both association studies and systems genetics analysis.

It is well known that genes do not act in isolation, but interact with one another to regulate complex traits. In addition, co-expressed genes usually have similar biological functions or are involved in same biochemical pathways. Therefore, building gene networks would provide an alternate way to identify potential regulators and gain insight into the underlying mechanisms of lipid metabolism (Stuart et al., 2003). To date, several algorithms have been developed to construct gene networks (Henry et al., 2014), and weighted gene co-expression network analysis (WGCNA) is the most widely used (Langfelder and Horvath, 2008). In addition to constructing gene networks, this method also allows one to summarize hub genes and module eigengenes (MEs). These can be used to subsequently identify trait-associated modules by performing “guilt-by-association” between phenotypes and eigengenes.

**Figure 1:**
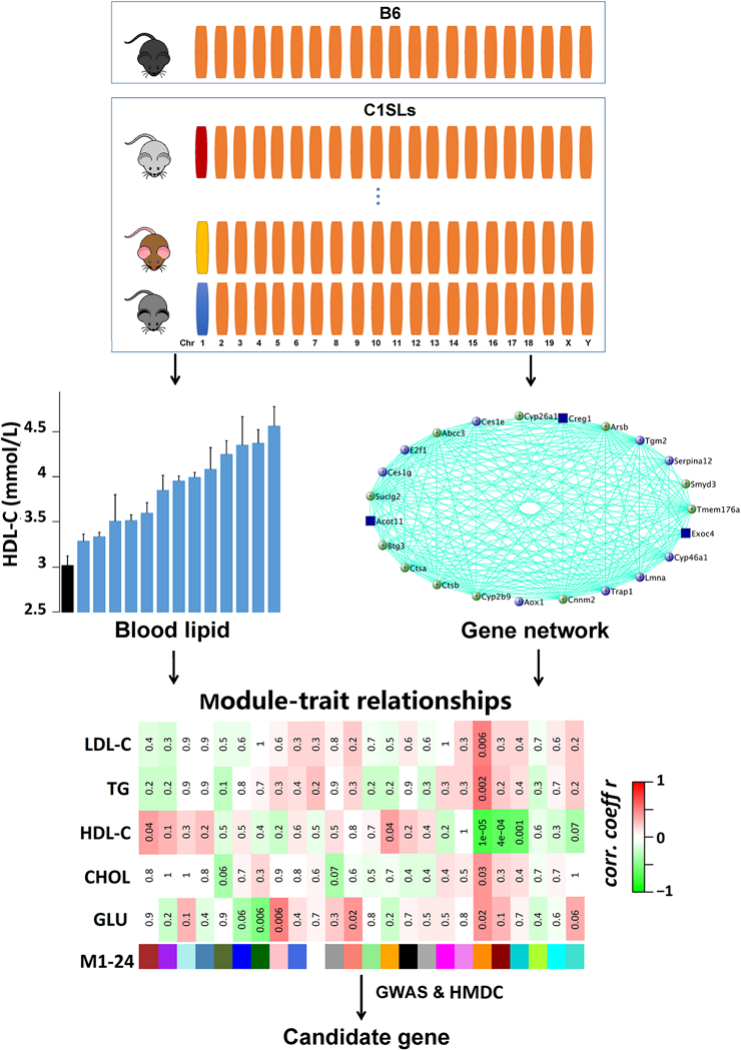
Schematic of the methodology. A total of 14 strains (13 C1SLs and one recipient strain B6) were involved in this study. The upper panel shows the characteristics of C1SLs genome background. Orange bars represent B6 chromosome while the others represent different donor chromosomes from wild mice. Blood lipid and fasting glucose levels were measured at 20 weeks of age. Liver gene co-expression network was constructed with WGCNA. The trait-associated modules were identified through testing the association between traits and MEs. The candidate genes were further dominated by integrating human GWAS and HMDC data.

Several studies have demonstrated that Chr 1 harbors multiple genetic loci that regulate plasma lipid and glucose levels (Orozco et al., 2009; Leduc et al., 2011). In order to identify the casual genes, we measured plasma lipid and fasting glucose levels in C1SLs and quantified transcriptome levels of liver with RNA-seq technique. By combining gene co-expression network analysis with human GWAS and gene functional annotation, several plasma lipid and glucose regulating candidate genes, especially those located on Chr 1, were identified (**Figure 1**).

## Materials and Methods

### Mice and Diet

All animal procedures were performed in accordance with guidelines of the Laboratory Animal Committee of Donghua University. 13 C1SLs and one B6 strain of adult male mice (an average of seven per strain; n = 97) were housed in a room maintained at 18–22°C with a 12-h light and 12-h dark cycle (6:00 A.M. to 6:00 P.M.). All animals were given a chow diet (M01-F25; Shanghai SLAC Laboratory Animal, Shanghai, China) for eight weeks, then fed with D12450B diet containing 4.3% fat, 19.2% protein, and 67.3% carbohydrate (Research Diets, New Brunswick, USA) until sacrificed by cervical dislocation at 20 weeks of age.

### Experiment Measurement

Blood was collected into 1.5-ml tubes with EDTA by retro-orbital bleeding from mice fasted for 4 h in the morning. Blood serum was separated by centrifugation at 2,500*g* for 15 min and frozen at −20°C until performing cholesterol enzymatic assays assay. Enzymatic assays for CHOL, HDL-C, LDL-C, TG, and glucose (GLU) were performed with biochemical blood analyzer (Hitachi 7180; Hitachi, Tokyo, Japan) by Sino-British SIPPR/B&K Lab Animal (Shanghai, China).

### RNA Isolation and Quality Control

RNA was extracted from liver tissues using RNAiso Plus reagent(TaKaRa Biotechnology, Dalian, China) according to the manufacturer’s protocol. RNA quality was analyzed using NanoDrop 2000c and Bioanalyzer. Samples with A260/A280 of 1.8–2.0 and RNA integrity number greater than 8 were subsequently used for sequencing library preparation.

### RNA Library Preparation and Sequencing

Twenty nine mRNA samples (two samples per strain except for strain LY) were used for RNA library preparation and sequencing. The poly(A) mRNA isolation was performed using Poly(A) mRNA Magnetic Isolation Module or rRNA removal Kit. The mRNA fragmentation and priming was performed using First Strand Synthesis Reaction Buffer and Random Primers. First strand cDNA was synthesized using ProtoScript II Reverse Transcriptase and the second-strand cDNA was synthesized using Second Strand Synthesis Enzyme Mix. The purified double-stranded cDNA by beads was then treated with End Prep Enzyme Mix to repair both ends and add a dA-tailing in one reaction, followed by a T-A ligation to add adaptors to both ends. Size selection of Adaptor-ligated DNA was then performed using beads, and fragments of ∼420 bp (with the approximate insert size of 300 bp) were recovered. Each sample was then amplified by PCR for 13 cycles using P5 and P7 primers, with both primers carrying sequences which can anneal with flow cell to perform bridge PCR and P7 primer carrying a six-base index allowing for multiplexing. The PCR products were cleaned up using beads, validated using an Qsep100 (Bioptic, Taiwan, China), and quantified by Qubit3.0 Fluorometer (Invitrogen, Carlsbad, USA). Then libraries with different indices were multiplexed and sequenced on Illumina X-ten instrument (Illumina, San Diego, USA) by GENEWIZ (Suzhou, China) according to the manufacturer’s instructions. Sequencing was carried out using a 2x150bp paired-end (PE) configuration.

### Reads Mapping and Quantification

Reads were aligned to the mouse reference genome (GRCm38) using Tophat2 (Kim et al., 2013) with default parameters. The Cufflinks program Cuffnorm (Trapnell et al., 2010) was used to generate tables of expression values (Fragments Per Kilobase of transcript per Million mapped reads, FPKM) which were normalized for library size based on GRCm38 gene annotation downloaded from iGenome (https://support.illumina.com/sequencing/sequencing_software/igenome.html). Expression data were further filtered to remove genes that had less than 1 FPKM in 20% or more samples and then log-transformed with log2 (FPKM+1).

### Weighted Gene Co-Expression Network Analysis (WGCNA)

Log2 transformed expression values were analyzed with WGCNA package in R (Langfelder and Horvath, 2008) to construct gene co-expression networks. Briefly, a correlation matrix was obtained by calculating pair-wise Pearson correlation coefficients between all genes across all samples. Then, a soft thresholding power β = 6 was chosen based on scale-free topology (R^2^ > 0.9) to generate weighted adjacency matrix. The adjacency matrix was further transformed into Topological Overlap Matrix which assesses transcript interconnectedness. Following this, a dissimilarity measure was calculated. Genes were aggregated into modules by hierarchical clustering based on Topological Overlap Matrix and further refined using the dynamic tree cut algorithm. ME is the first principal component of a given module, and it was used to evaluate the module membership, which assessed the importance of genes in the network.

### Candidate Gene Analysis Using Publicly Available Resources

We prioritized the candidates using the following public resources:

1. **Human–Mouse: Disease Connection (HMDC)**. This resource included mouse and human gene-trait relationships from several databases, including Mouse Genome Informatics database (MGI), National Center for Biotechnology Information (NCBI), Online Mendelian Inheritance in Man (OMIM), and the Human Phenotype Ontology (HPO).
2. **Human GWAS**. Human GWAS for plasma lipid and fasting glucose levels were obtained from GRASP (https://grasp.nhlbi.nih.gov) (Leslie et al., 2014) and GWAS Catalog (https://www.ebi.ac.uk/gwas/) (Macarthur et al., 2016). GRASP includes available genetic association studies with p value <0.05. GWAS Catalog collects SNP-trait associations with p value <1 × 10^−6^. In the present study, mapped genes or genes nearest to the marker with p value < 1 × 10^−4^ were used to looking for overlap with module gene lists.
3. **Gene expression atlas across mouse tissue**. Gene expression profiles for 22 mouse tissues, which were generated by the Mouse ENCODE project using RNA-seq (Yue et al., 2014), were queried from NCBI (https://www.ncbi.nlm.nih.gov/). We define genes with “high liver expression” as those with an expression level in liver greater than threefold of the mean expression value across the 22 tissues.

### Identification of Genetic Variants for the Candidate Genes

Genetic variants between C1SLs and B6 were identified with whole genome sequencing as previously described (Xu et al., 2016). Variant annotation was performed using Variant Effect Predictor (Mclaren et al., 2016).

## Results

### C1SLs Exhibits Broad Phenotypic Variability in Plasma Lipid and Fasting Glucose Levels

**Figure 2:**
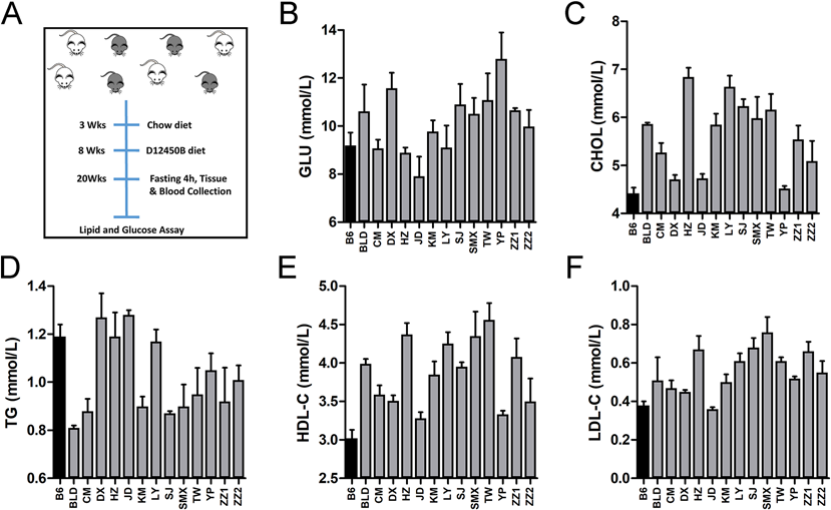
Phenotype distributions across C1SLs and B6. **(A)** Schematic of traits collection **(B**–**F)** Bar plot of plasma lipid and fasting glucose levels across C1SLs and B6 (mean + standard errors). Each bar represents one strain, and the black corresponds to the recipient strain B6.

In this study, plasma lipid (CHOL, HDL-C, LDL-C, and TG) and fasting glucose levels of 13 C1SLs and one recipient strain B6 were examined using enzymatic assays (**Figure 2A**). Assay results demonstrate broad phenotypic variability with fold change 1.62 in GLU, 1.55 in CHOL, 1.51 in HDL-C, 2.11 in LDL-C and 1.58 in TG (**Figures 2B**–**F** and **Supplementary Data S1**). Compared to the C1Sls, recipient strain B6 showed relatively low levels of GLU, CHOL, HDL-C, and LDL-C and a high level of TG.

### WGCNA Identifies Several Modules Significantly Associated With Plasma Lipid and Fasting Glucose Levels

We carried out high throughput RNA-seq using Ilumina X-ten platform to comprehensively quantify the gene abundance of liver tissue for 29 samples. A total of ~2.3 billion reads were obtained, ranging from 26 million to 0.42 billion per sample (**Supplementary Data S2**). The raw reads were mapped onto the mouse genome with an average of 80% of the read pairs that are properly assigned. Gene expression levels were generated and normalized with Cuffnorm program. Further filtration was applied (See *Materials and Methods*), which resulted in 10,525 genes for subsequent analysis (**Supplementary Data S3**).

**Figure 3:**
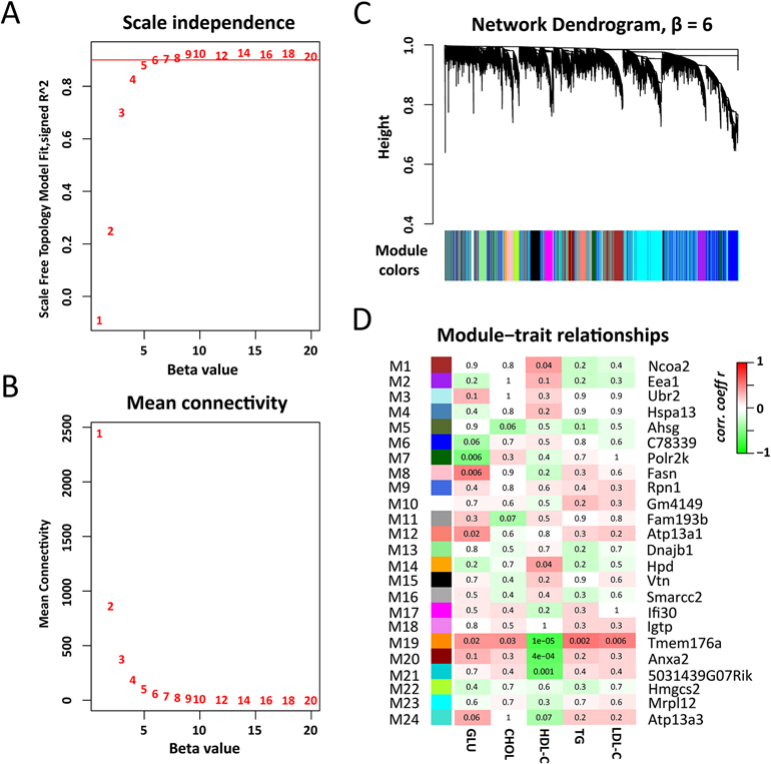
Weighted gene co-expression network analysis of liver transcriptomes. **(A)** The soft thresholding index R^2^ (y-axis) as a function of different thresholding power β (x-axis). **(B)** Mean connectivity (y-axis) as a function of the power β (x-axis). **(C)** Twenty four co-expression modules were identiﬁed from the liver RNA-seq dataset. WGCNA cluster dendrogram groups genes (n = 10,520) measured across C1SLs and its recipient strain B6 liver into distinct gene modules (M1–24) deﬁned by dendrogram branch cutting. **(D)** Module-trait associations. Each row corresponds to a module column to a trait. Each cell contains the corresponding correlation and p-value. The table is color-coded by correlation according to the color legend. The hub genes were indicated aside each module.

To identify regulatory genes for plasma lipid and glucose levels. We constructed gene co-expression networks using WGCNA. With the soft-thresholding power parameter (β = 6) determined by the scale-free topology (**Figures 3A**, **B**), a total of 24 modules (after excluding module gray) were identified (**Figures 3C**, **D** and **Supplementary Data S3**). The module size (i.e., the total number of genes in a module) varies significantly, ranging from 39 genes in module M5 to 2,141 genes in module M24. Among those modules, M19 (83 genes) is significantly associated with all five traits (**Figure 3D**), while M1 (930 genes), M14 (99 genes), M20 (389 genes), M21 (117 genes) are significantly linked to TG level and M7 (491 genes), M8 (311 genes), and M12 (247 genes) are associated with fasting glucose level (**Figure 3D**).

### Gene Prioritizing of Trait-Associated Modules

**Figure 4:**
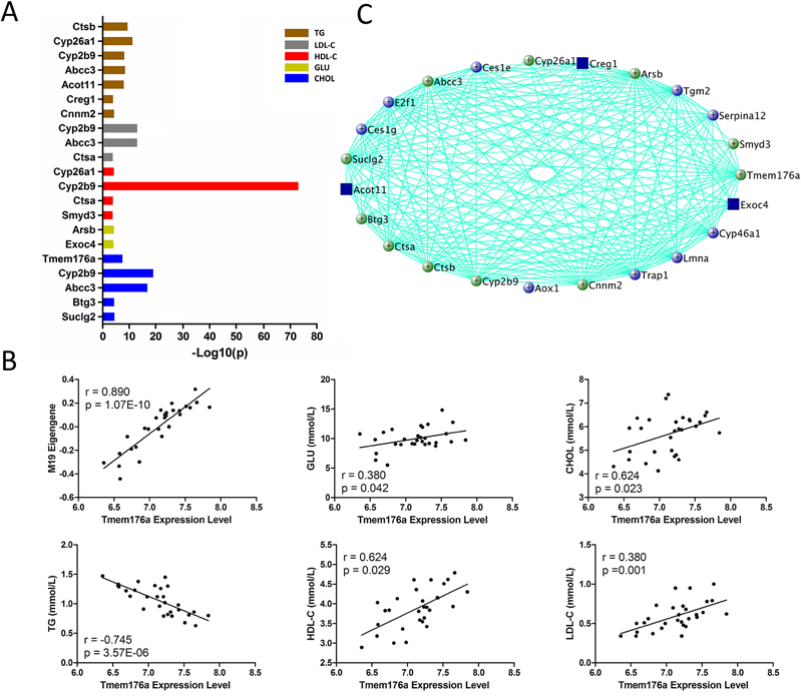
Gene prioritization for module M19. **(A)** Human GWAS overlapping genes for module M19. Genes with GWAS p value <1 × 10^−4^ for blood lipid and fasting glucose levels were retrieved from GRASP and GWAS Catalog. **(B)** Correlations between module M19 hub gene *Tmem176a* and MEs and traits. **(C)** Gene subnetwork for module M19. Green circles represent genes overlapping with human GWAS; blue circles represent genes functionally validated in mouse models; both functionally validated and GWAS overlapping genes are marked with blue rectangles.

M19 is the module most significantly associated with all of the traits. There are 83 genes in this module, and 74 are significantly correlated (p < 0.05) with these traits and MEs simultaneously (**Supplementary Data S4**). Gene ontology (biological process) enrichment analysis revealed that these genes are significantly enriched in lipid metabolism and gluconeogenesis regulation (**Supplementary Data S5**). In addition, 14 genes are found in human GWAS with p value <1 × 10^−4^ (**Figure 4A** and **Supplementary Data S6**) and 11 genes are known regulators for blood lipid or glucose metabolism (**Figure 4C**). Four of them, *Creg1*, *Abcc3*, *Cyp2b9*, and *Cyp26a1*, are highly expressed in liver (**Supplementary Figure 1**). More importantly, the module hub gene, *Tmem176a*, is significantly correlated with blood lipid levels (**Figure 4B** and **Supplementary Data S4**) and have been mapped to CHOL in human GWAS with a p value of 2 × 10^−8^ (**Supplementary Data S6**).

**Figure 5:**
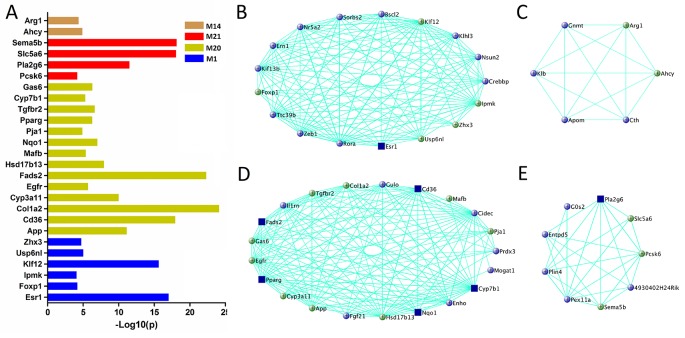
Gene prioritization for TG-associated modules. **(A)** Human GWAS overlapping genes for TG-associated modules. Genes with GWAS p value <1 × 10^−4^ of TG level were retrieved from GRASP and GWAS Catalog. **(B**–**E)** Gene subnetworks for module M1, M14, M20, and M21. The coloring scheme is same as **Figure 4C**.

For other modules associated with TG level (M1, M14, M20, and M21), 505 genes are significantly correlated with TG and their MEs simultaneously (**Supplementary Data S7**), 26 genes are found in human GWAS (**Figure 5A** and **Supplementary Data S6**), and 40 are essential for TG metabolism (**Figures 5B**–**E**). In addition, six genes, *Egfr*, *Hsd17b13*, *Cyp3a11*, *Arg1*, *Fads2*, and *Ahcy*, are highly expressed in liver (**Supplementary Figure 1**).

**Figure 6:**
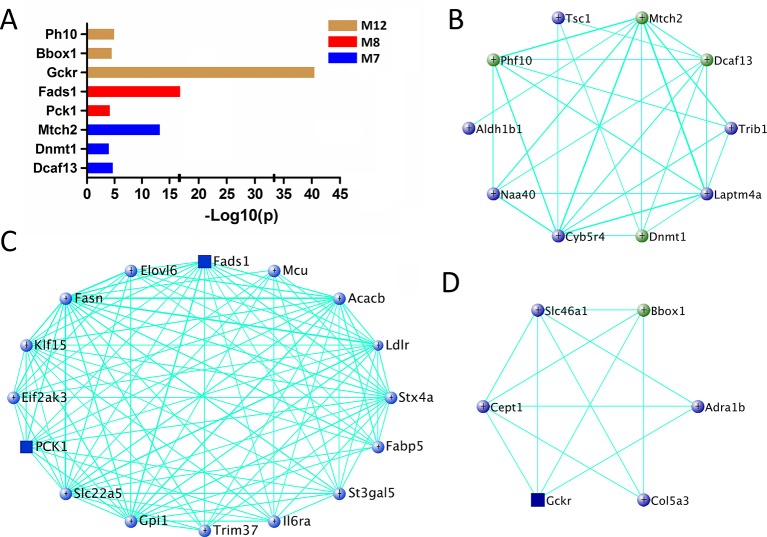
Gene prioritization for fasting glucose-associated modules. **(A)** Human GWAS overlapping genes for fasting glucose-associated modules. Genes with GWAS p value <1 × 10^−4^ of fasting glucose level were retrieved from GRASP and GWAS Catalog. **(B**–**D)** Gene subnetworks for module M7, M8, and M12. The coloring scheme is same as **Figure 4C**.

For other modules associated with fasting glucose level (M7, M8, and M12), 377 genes are significantly associated with fasting glucose and their MEs simultaneously (**Supplementary Data S8**). Among them, eight genes are found in human in GWAS (**Figure 6A** and **Supplementary Data S6**), and 27 genes are known glucose metabolism regulators (**Figures 6B**–**D**). Furthermore, three of them, *Pck1*, *Fads1*, and *Gckr*, are highly expressed in liver (**Supplementary Figure 1**).

### Prioritizing Causative Genes on Chr 1

Due to the fact that C1SLs only differ from B6 strain by one chromosome (**Figure 1**), we believe the phenotypic differences are partly driven by the genetic variations on Chr 1. In this study, a total of 38 genes in the trait-associated modules were found to be located on Chr 1. Of which, 35 harbor missense single nucleotide polymorphisms (SNPs), and all have 3’ or 5’ UTR variants (**Table 1**). In addition, several genes have been associated with the traits in mouse models, including *Creg1* and *Aox1* in module M19; *Phlpp1*, *Nr5a2*, *Rnf149*, *Ncoa2*, and *Abl2* in module M1; *Mogat1*, *Igfbp2*, and *Col3a1* in module M20; *G0s2*, *Crp*, and *Ppox* in module M21, M7, and M12, respectively.

**Table 1:** Lists of the module-trait associated genes on Chr 1.

Gene symbol	Entrez ID	Chr	Start	End	Module	Associated traits	# Missense SNP	# UTR SNP
***Creg1***	**433375**	**1**	**165763746**	**165775308**	**M19**	**CHOL, HDL, LDL, TG, GLU**	**1**	**118**
*Atic*	108147	1	71557150	71579631	M19	CHOL, HDL, LDL, TG, GLU	4	31
***Aox1***	**11761**	**1**	**58029931**	**58106413**	**M19**	**CHOL, HDL, LDL, TG, GLU**	**18**	**5**
*Smyd3*	69726	1	178951960	179518041	M19	CHOL, HDL, LDL, TG, GLU	10	152
*Igsf8*	140559	1	172261641	172319841	M19	CHOL, HDL, LDL, TG, GLU	7	29
*Inpp4a*	269180	1	37299865	37410736	M1	TG	39	98
***Phlpp1***	**98432**	**1**	**106171752**	**106394250**	**M1**	**TG**	**9**	**20**
*Eprs*	107508	1	185363044	185428360	M1	TG	14	7
*Ino80d*	227195	1	62958418	63114667	M1	TG	36	231
***Nr5a2***	**26424**	**1**	**136842571**	**136960448**	**M1**	**TG**	**5**	**93**
***Rnf149***	**67702**	**1**	**39551296**	**39577405**	**M1**	**TG**	**2**	**15**
***Ncoa2***	**17978**	**1**	**13139105**	**13374083**	**M1**	**TG**	**6**	**59**
***Abl2***	**11352**	**1**	**156558786**	**156649568**	**M1**	**TG**	**5**	**113**
*Kmo*	98256	1	175620381	175662116	M1	TG	20	105
*Myo1b*	17912	1	51749765	51916071	M1	TG	4	78
*Wdr26*	226757	1	181173228	181211552	M1	TG	3	100
*Rabgap1l*	29809	1	160219174	160793211	M1	TG	16	62
*Sde2*	208768	1	180851127	180868113	M1	TG	21	28
*Gm38394*	NA	1	133619940	133661318	M1	TG	6	112
*1700034P13Rik*	73331	1	9747648	9791924	M1	TG	0	0
*Etnk2*	214253	1	133363572	133380336	M14	TG	12	62
***Mogat1***	**68393**	**1**	**78510991**	**78538173**	**M20**	**TG**	**0**	**3**
***Igfbp2***	**16008**	**1**	**72824503**	**72852474**	**M20**	**TG**	**0**	**5**
*Cps1*	227231	1	67123026	67231259	M20	TG	1	11
***Col3a1***	**12825**	**1**	**45311538**	**45349706**	**M20**	**TG**	**2**	**10**
*Rpl28-ps1*	100042670	1	128038569	128038982	M20	TG	0	0
*Aox3*	71724	1	58113130	58200698	M20	TG	14	35
*2810459M11Rik*	72792	1	86045863	86055456	M20	TG	6	56
***G0s2***	**14373**	**1**	**193272161**	**193273217**	**M21**	**TG**	**0**	**7**
*Mrps9*	69527	1	42851233	42905683	M21	TG	5	22
***Crp***	**12944**	**1**	**172698055**	**172833031**	**M7**	**GLU**	**2**	**10**
*Ppil3*	NA	1	82233112	82235933	M7	GLU	6	40
*Tmem131*	56030	1	36792194	36943666	M7	GLU	16	19
*Tmem185b*	226351	1	119526160	119528983	M7	GLU	1	26
*Tex30*	75623	1	44086613	44102441	M7	GLU	0	24
*9430016H08Rik*	70225	1	58430994	58445486	M7	GLU	0	0
*Gm9747*	68115	1	57406328	57417953	M7	GLU	1	10
***Ppox***	**19044**	**1**	**171275990**	**171281186**	**M12**	**GLU**	**9**	**10**

Bold highlighted genes are known functional genes.

## Discussion

Recent work has demonstrated that gene co-expression network analysis is a powerful way to associate genes with specific phenotypes. Here, WGCNA was applied to investigate liver transcriptomes of C1SL mice. A total of 24 modules were identified, with module M19 being significantly associated with blood lipid and glucose levels (**Figure 3D**). Searching MGI database revealed that 13% (11 out of 84) of M19 genes are involved in blood lipid or glucose metabolism (**Figure 4C**), such as acyl-CoA thioesterase 11 (*Acot11*) (Zhang et al., 2012), cellular repressor of E1A-stimulated genes 1 (*Creg1*) (Tian et al., 2017), carboxylesterase 1E (*Ces1e*) (Wei et al., 2010), carboxylesterase 1G (*Ces1g*) (Wei et al., 2010), and lamin A (*Lmna*) (Arimura et al., 2005). This suggests that glucose and lipid metabolism share common genetic architecture (Parhofer, 2015). We also identified several TG (M1, M14, M20, and M21) and fasting glucose (M7, M8, and M12) associated modules. These modules include several known functional genes (**Figures 5** and **6**), such as peroxisome proliferator activated receptor gamma (*Pparg*) (Heikkinen et al., 2009), cell death-inducing DFFA-like effector c (*Cidec*) (Toh et al., 2008), monoacylglycerol O-acyltransferase 1 (*Mogat1*) (Agarwal et al., 2016), glucokinase regulatory protein (*Gckr*) (Farrelly et al., 1999), and phosphoenolpyruvate carboxykinase 1(*Pck1*) (Hakimi et al., 2005). Since co-expressed genes are assumed to be involved in interconnected biological pathways (Weirauch, 2011), we believe other genes, along with the known functional genes in the trait-associated modules, also serve regulatory roles in glucose and lipid metabolism.

Human GWAS in relation to blood lipid and glucose metabolism have identified hundreds of associated genes (Kathiresan et al., 2007; Kathiresan et al., 2008; Dupuis et al., 2010; Willer et al., 2013a; Hwang et al., 2015; Siewert and Voight, 2018). However, most variants identified so far only explain a small portion of phenotypic variance, leaving the majority of heritability unexplained (Manolio et al., 2009). The inability to uncover the remaining spectrum of variance is related to multiple factors, including sample size, genetic structure, rare variants, and gene-gene interactions (Manolio et al., 2009; Parker and Palmer, 2011). In addition, stringent thresholds of p-value with high multiple testing corrections is also believed to exclude many positive signals (Lee et al., 2011; Lee and Lee, 2018). Joint analysis of human GWAS and mouse genetics would help to “rescue” some of the ‘missing’ heritability (Parker and Palmer, 2011; Ashbrook et al., 2014; Ashbrook et al., 2015; Wang et al., 2016). In the present study, we identified 48 genes in the trait-associated modules which have been reported in human GWAS with p value <1 × 10^−4^. Among them, several genes not only achieved GWAS significance threshold (p value <5 × 10^−8^), but also functionally validated in mouse models, including acyl-CoA thioesterase 11 (*Acot11*) (Asselbergs et al., 2012; Zhang et al., 2012), estrogen receptor 1(*Esr1*) (Ohlsson et al., 2000; Asselbergs et al., 2012), Cd36 molecule (*Cd36*) (Goudriaan et al., 2003; Asselbergs et al., 2012), fatty acid desaturase 2 (*Fads2*) (Stroud et al., 2009; Teslovich et al., 2010), phospholipase A2, group VI (*Pla2g6*) (Zhang et al., 2013; Spracklen et al., 2017), glucokinase regulatory protein (*Gckr*) (Farrelly et al., 1999; Scott et al., 2012), and fatty acid desaturase 1 (*Fads1*) (Scott et al., 2012). Furthermore, we also found several functionally validated genes with modest GWAS p values, such as cellular repressor of E1A-stimulated genes 1 (*Creg1*) (Saxena et al., 2007; Tian et al., 2017), cytochrome P450 family 7 subfamily b polypeptide 1 (*Cyp7b1*) (Li-Hawkins et al., 2000; Del-Aguila et al., 2014), NAD(P)H dehydrogenase quinone 1 (*Nqo1*) (Gaikwad et al., 2001; Asselbergs et al., 2012), peroxisome proliferator activated receptor gamma (*Pparg*) (Heikkinen et al., 2009; Teslovich et al., 2010), and phosphoenolpyruvate carboxykinase 1(*Pck1*) (Hakimi et al., 2005; Dupuis et al., 2010). Although the function of other genes (GWAS p value > 5 × 10^−8^) in plasma lipid or glucose metabolism remain unclear, they are possible candidates based on the genetic evidence from our results (**Figures 4**–**6**). Therefore, we believe that by intergrating human GWAS and mouse genetics studies, it is possible to identify more functional genes and uncover part of the ‘missing’ heritabilities caused by stringent statistical thresholds in human GWAS.

C1SLs are aimed to identify genes associated with complex traits on Chr 1 by performing association studies or systems genetics analysis. However, the current study only included 13 C1SLs and one recipient strain B6 and performing association studies in such a small number of strains could result in many false positives due to the low statistical power (Flint and Eskin, 2012). Therefore, we used the systems genetics strategy, gene co-expression network analysis, to prioritize the novel candidate genes-especially those located on Chr 1. A total of 38 Chr 1 genes are found in the eight trait-associated modules with an average of 4.75 genes in each. This number is far less than QTL genes identified by linkage studies in F2 mouse segregation population or association studies in mouse reference populations (Buchner and Nadeau, 2015). In addition, we found at least one gene for each module that has been implicated in regulation of plasma lipid or glucose metabolism (**Table 1**). Therefore, this approach could allow for identification of functional genes (Chr 1) more efficiently than using previous methods and mouse population.

In summary, we identified eight gene networks associated with blood lipid and glucose levels by performing gene co-expression network analysis in C1SL mice population. Further joint analysis of human GWAS resulted in 48 candidate functional genes. In addition, 38 genes on Chr 1, including 13 well characterized genes, are prioritized as causative genes. However, these genes still need further studies to illustrate their potential functional roles. With the development of other C1SLs and further achiving of sequencing data, Co-expression network analysis on C1SLs can provide us a new avenue for identifying other causative genes for complex traits on Chr 1.

## Data Availability Statement

All raw reads were submitted to NCBI Sequence Read Archive with the accession number SRP198324.

## Ethics Statement

All animal procedures were performed in accordance with guidelines of the Laboratory Animal Committee of Donghua University.

## Author Contributions

JX conceived and supervised the study. MW and FX performed the experiment and data analysis. SH helped to collect RNA. MW and FX wrote the manuscript. JC, YZ, KL, and HX edited the manuscript. All authors read and approved the final version of the manuscript.

## Funding

This work was supported by National Natural Science Foundation of China (Grant no. 31772550), the Key Project of Science and Technology Commission of Shanghai Municipality (No. 16140901300, 16140901302).

## Conflict of Interest

The authors declare that the research was conducted in the absence of any commercial or financial relationships that could be construed as a potential conflict of interest.

## Supplementary Material

Supplementary Data S1 Phenotype information.

Supplementary Data S2 Summarization of RNA-seq.

Supplementary Data S3 Gene-module lists & Expression values.

Supplementary Data S4 M19 module’s GXP and GXM relationships.

Supplementary Data S5 GO enrichment of the M19 module genes.

Supplementary Data S6 Lists of the GWAS signals overlapped with the trait-associated module genes.

Supplementary Data S7 Other TG-associated modules’ GXP & GXM relationships.

Supplementary Data S8 Other fasting glucose-associated modules’ GXP & GXM relationships.

Supplementary Figure 1 Heatmap of the candidate gene expression levels across the 22 mouse tissues.
