# Supplementary figures and images for: Candidate Regulators of Dyslipidemia in Chromosome 1 Substitution Lines Using Liver Co-Expression Profiling Analysis
Source: Front Genet. 2020 Jan 9;10:1258. doi: 10.3389/fgene.2019.01258 (PMC6962132; doi:10.3389/fgene.2019.01258)

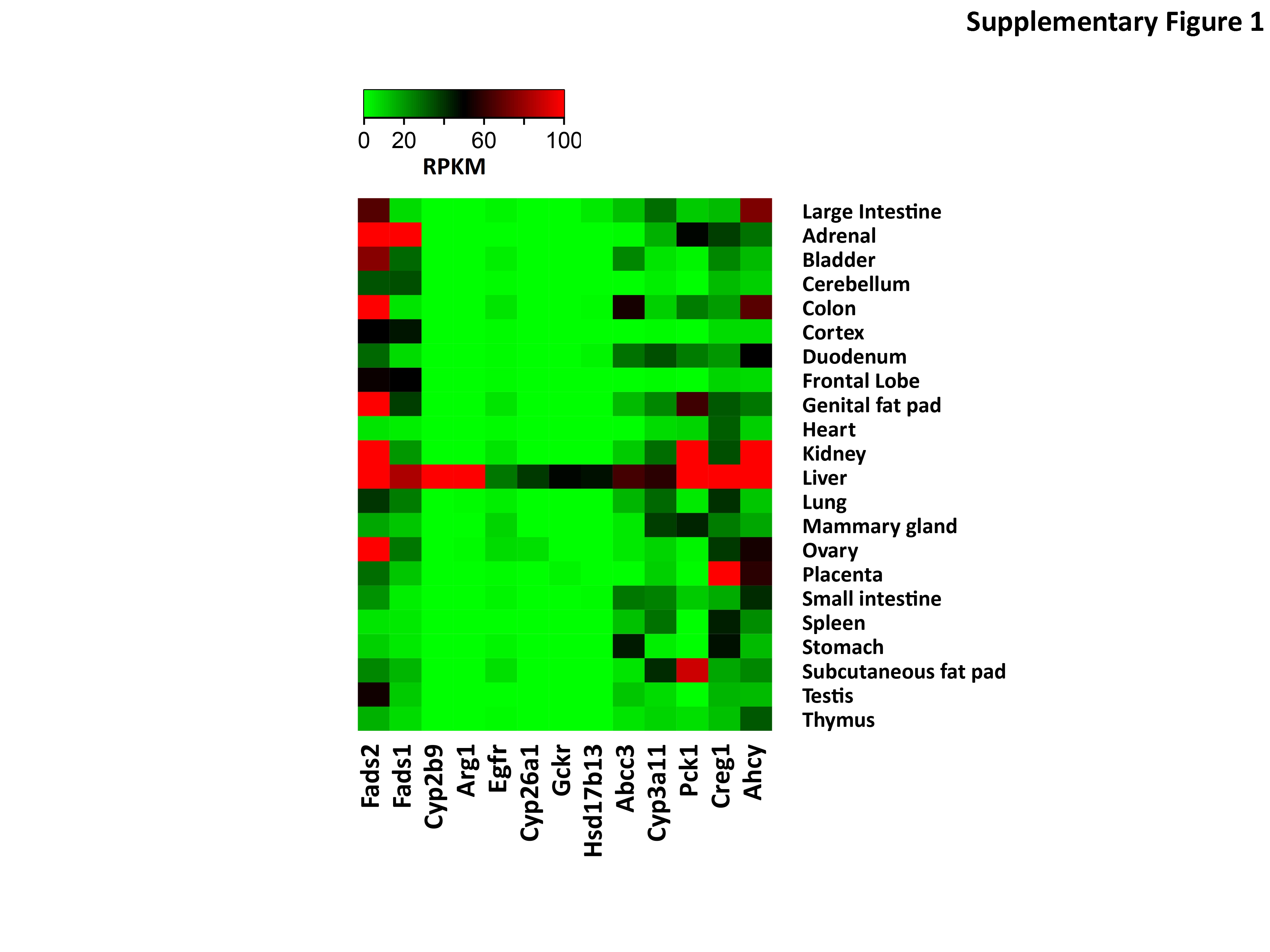

Supplement: Supplementary Figure 1 — Heatmap of the candidate gene expression levels across the 22 mouse tissues. [file Image_1.jpeg]
